# Correction: Therapeutic Non-Toxic Doses of TNF Induce Significant Regression in TNFR2-p75 Knockdown Lewis Lung Carcinoma Tumor Implants

**DOI:** 10.1371/journal.pone.0101647

**Published:** 2014-06-24

**Authors:** The PLOS ONE Staff

## Notice of Republication

This article was republished on June 5, 2014, to replace an incorrectly published version. During the typesetting process, an equation in the Results section was removed from the XML and PDF of the article. Please download this article again to view the correct version. The originally published, uncorrected article and the republished, corrected article are provided here for reference.

## Supporting Information

File S1 Originally published, uncorrected article.
(PDF)

File S2 Republished corrected article.
(PDF)
