# Correction: Therapeutic Non-Toxic Doses of TNF Induce Significant Regression in TNFR2-p75 Knockdown Lewis Lung Carcinoma Tumor Implants

**DOI:** 10.1371/journal.pone.0144213

**Published:** 2015-11-30

**Authors:** Sharath P. Sasi, Sanggyu Bae, Jin Song, Aleksandr Perepletchikov, Douglas Schneider, Joseph Carrozza, Xinhua Yan, Raj Kishore, Heiko Enderling, David A. Goukassian

In the Funding section, the grant number NNJ10ZSA001N from the funder National Aeronautic and Space Administration has been updated by the funder. The updated grant number is: NNX11AD22G.
