# Supplementary material for: Correction: Therapeutic Non-Toxic Doses of TNF Induce Significant Regression in TNFR2-p75 Knockdown Lewis Lung Carcinoma Tumor Implants
Source: PLoS One. 2014 Jun 24;9(6):e101647. doi: 10.1371/journal.pone.0101647 (PMC4069119; doi:10.1371/journal.pone.0101647)
Supplement: File S2 — Republished corrected article. (PDF) [file pone.0101647.s002.pdf]

PLOS ONE

Therapeutic Non-Toxic Doses of TNF Induce Significant Regression in TNFR2-p75 Knockdown Lewis Lung Carcinoma Tumor Implants

Sharath P. Sasi[1], Sanggyu Bae[1,2], Jin Song[1], Aleksandr Perepletchikov[2,3], Douglas Schneider[2,3], Joseph Carrozza[2,3], Xinhua Yan[1,3], Raj Kishore[4], Heiko Enderling[5], David A. Goukassian[1,3]*

1 Cardiovascular Research Center, GeneSys Research Institute, Boston, Massachusetts, United States of America, **2** Departments of Medicine and Pathology, Steward St. Elizabeth' Medical Center, Boston, Massachusetts, United States of America, **3** Tufts University School of Medicine, Boston, Massachusetts, United States of America, **4** Feinberg Cardiovascular Institute, Northwestern University, Chicago, Illinois, United States of America, **5** Integrated Mathematical Oncology, Moffitt Cancer Center and Research Institute, Tampa, Florida, United States of America

Abstract

Tumor necrosis factor-alpha (TNF) binds to two receptors: TNFR1/p55-cytotoxic and TNFR2/p75-pro-survival. We have shown that tumor growth in p75 knockout (KO) mice was decreased more than 2-fold in Lewis lung carcinoma (LLCs). We hypothesized that selective blocking of TNFR2/p75 LLCs may sensitize them to TNF-induced apoptosis and affect the tumor growth. We implanted intact and p75 knockdown (KD)-LLCs (>90%, using shRNA) into wild type (WT) mice flanks. On day 8 post-inoculation, recombinant murine (rm) TNF-α (12.5 ng/gr of body weight) or saline was injected twice daily for 6 days. Tumor volumes (tV) were measured daily and tumor weights (tW) on day 15, when study was terminated due to large tumors in LLC+TNF group. Tubular bones, spleens and peripheral blood (PB) were examined to determine possible TNF toxicity. There was no significant difference in tV or tW between LLC minus (-) TNF and p75KD/LLC-TNF tumors. Compared to 3 control groups, p75KD/LLC+TNF showed >2-5-fold decreases in tV ($p<0.001$) and tW ($p<0.0001$). There was no difference in tV or tW end of study vs. before injections in p75KD/LLC+TNF group. In 3 other groups tV and tW were increased 2.7-4.5-fold ($p<0.01$, $p<0.0002$ and $p<0.0001$). Pathological examination revealed that 1/3 of p75KD/LLC+rmTNF tumors were 100% necrotic, the remaining revealed 40-60% necrosis. No toxicity was detected in bone marrow, spleen and peripheral blood. We concluded that blocking TNFR2/p75 in LLCs combined with intra-tumoral rmTNF injections inhibit LLC tumor growth. This could represent a novel and effective therapy against lung neoplasms and a new paradigm in cancer therapeutics.

Citation: Sasi SP, Bae S, Song J, Perepletchikov A, Schneider D, et al. (2014) Therapeutic Non-Toxic Doses of TNF Induce Significant Regression in TNFR2-p75 Knockdown Lewis Lung Carcinoma Tumor Implants. PLoS ONE 9(3): e92373. doi:10.1371/journal.pone.0092373

Editor: Rajasingh Johnson, University of Kansas Medical Center, United States of America

Received January 14, 2014; **Accepted** February 19, 2014; **Published** March 24, 2014

Copyright: © 2014 P Sasi et al. This is an open-access article distributed under the terms of the Creative Commons Attribution License, which permits unrestricted use, distribution, and reproduction in any medium, provided the original author and source are credited.

Funding: This material is based upon work supported by the National Aeronautic and Space Administration under Grant No. NNJ10ZSA001N to D.A.G., and in part by grants from AHA 10GRNT4710003 and NHLBI HL106098 to X.Y. and NIH HL091983 to R.K. The funders had no role in study design, data collection and analysis, decision to publish, or preparation of the manuscript.

Competing Interests: The authors have declared that no competing interests exist.

* Email: david.goukassian@tufts.edu

Introduction

Despite recent advances in the treatment of lung malignancies, lung cancer is still the most common cause of cancer-related deaths in humans [1,2]. Although standard treatments for these tumors advanced, the five year survival after diagnosis remains low. Importantly, tumors quickly develop resistance to therapeutic drugs or could harbor chemoresistant clones from the beginning [3,4]. Therefore, the development of novel therapeutic strategies is mandated for the treatment of these types of cancer.

Tumor necrosis factor-alpha (TNF) has been implicated in almost all steps of tumorigenesis. TNF induces its effects by binding two distinct receptors, TNFR1/p55 and TNFR2/p75 [5,6]. Expression of p55 is constitutive in most of the cells, whereas expression of p75 appears to be inducible [7]. Due to significant differences in the cytoplasmic domain, it has been postulated that TNF receptors trigger distinct signaling pathways upon interaction with the ligand TNF [8]. In agreement with this concept, activation of the caspases cascade and subsequent induction of apoptosis by TNF is an exclusive feature of p55 activation through signaling via a well-defined death domain, which is absent in p75 receptor signaling [9,10]. Only few specific signaling pathways of p75 have been elucidated, especially within the context of tumorigenesis and endothelial cell (ECs) biology [11–13].

All currently existing anticancer therapies involving TNF are targeted towards the ligand, TNF itself, which includes administration of very high doses of exogenous TNF for melanoma treatment [14–16] or use of soluble receptors and/or various forms of TNF antibodies (Remicade, Humira) and a soluble TNF receptor fusion protein (Enbrel) [17,18]. Both, monoclonal antibodies and soluble receptors are mediating their effects via binding with high specificity and affinity to soluble and membrane-bound TNF to block the interaction of TNF with, both, p55 and p75 receptors [17–19]. Therefore, TNF-based treatments in use or being developed are all based on either inhibition of TNF bioavailability or local perfusion of cancerous tissue with very high doses of TNF. In spite of having considerable

anti-tumor effect, these approaches have significant side effects such as septic shock-like syndrome, systemic inflammatory response, and excessive non-tumor tissue necrosis [15,16] that limit their use. To date, TNF-based treatments do not take into consideration the possibility of selective inhibition of p75 TNF receptor signaling pathways.

Our previously published findings in mouse lung and melanoma tumor models in p75 knockout (KO) murine model suggest that the absence/inhibition of p75 signaling in tumor tissue in vivo may deliver a "double hit" [13]. The experimental support for this entirely novel treatment approach and cancer growth control originates from our published data which indicate that absence of TNFR2/p75 in the host tissue of p75 knockout (KO) mice inhibited >50% growth of implanted Lewis lung carcinoma 1 (LLC) cells and metastatic B16 mouse melanoma cells (B16). Dwarfing the p75 receptor signaling pathways affects survival and function of both ECs and tumor cells, while continuous increasing levels of TNF in tumor tissue also have a "self-destructive" effect via signaling through the remaining cytotoxic TNFR1/p55 pathways. Therefore our current studies were geared towards selective inhibition of TNF-TNFR2 signaling axis as means for augmentation of tumor apoptosis and inhibition of tumor vascularity. This could represent a promising novel paradigm in cancer treatment.

Our hypothesis is that p75 is essential for tumor angiogenesis and survival, which if true has very important implications for tumor biology and development of more effective therapy since the p75 receptor is "drugable" through, for example, a blocking/neutralizing antibody. To the best of our knowledge our study is the first attempt to use selective inhibition of TNFR2/p75 for cancer treatment. The development of novel therapy based on selective inhibition of signaling via p75 could be an effective anti-angiogenic and pro-apoptotic mono-therapy, as well as a part of combination anti-cancer therapy that will help to sensitize tumor cells and tumor ECs to cytotoxic effects of conventional treatments such as chemotherapy [3] or radiation [12], thereby improving the outcome and decreasing toxicity and mortality.

Materials and Methods

Experimental animal model

Eight to twelve weeks old male WT C57BL/6J mice were purchased from Jackson Laboratory. All animals were handled in accordance with the guidelines set and approved by the GeneSys Research Institute (formerly known as Steward Research and Specialty Projects Corporation) Institutional Animal Care and Use Committee (IACUC) at Steward St Elizabeth's Medical Center of Boston. Any animal in this study found to exhibit severe or irreversible symptoms of infection (contamination of the tumor site with bacteria) or pain and stress (limited mobility, reduced consumption of food and water, weight loss of 15% or more) was euthanized immediately by Pentobarbital based euthanasia solution 200 mg/Kg intraperitoneal (i.p). This method is consistent with the recommendation of the Panel on Euthanasia of the American Veterinary Medical Association *Guidelines on Euthanasia.*

Cell culture

Mouse Lewis lung carcinoma (LLC1) cells were obtained from ATCC and maintained in high glucose DMEM media (Life Technologies, Grand Island, NY) supplemented with 10% fetal bovine serum (Atlanta Biologicals, Flowery Branch, GA), 100 units/ml penicillin/100 μg/ml streptomycin/0.25 μg/ml (Life Technologies) and 5 mM Sodium pyruvate solution (Sigma, St. Louis, MO).

shRNA plasmid DNA preparation and purification

SureSilencing shRNA plasmid kit for mouse p75 receptor (SuperArray, Valencia, CA) consisting of four target plasmids for p75 receptor and one negative plasmid were separately transformed by using 3 μl of each stock plasmid separately in XL-1 Blue competent E.Coli cells (Stratagene, La Jolla, CA) as per manufacturer protocol. Each transformed plasmid was used to initiate a separate starter mini culture for verification and quality control of plasmid vector and mega culture for generation of purified shRNA plasmid as per the protocol established earlier in our laboratory [13].

Stable LLC transfection using shRNA plasmids and geneticin selection of p75 knockdown LLCs

Purified shRNA plasmids were used to stably transfect LLCs to knockdown p75 receptor using four single plasmids and eight different plasmid combinations of these single plasmids along with negative control plasmid separately using Effectene transfection reagent (Qiagen, Valencia, CA) following manufacturer's protocol as detailed and optimized in our lab earlier [13]. Transfected LLCs were passaged and propagated in geneticin selective medium (600 μg/ml) along with non-transfected LLC as control for 6 days until colonies appeared in transfected LLC dish and control dish had no surviving cells [13]. RNA isolated from stably transfected cells were used to analyze p75 receptor knockdown (KD) levels by qRT-PCR [13]. LLCs with ≥90% p75 receptor knockdown (p75KD/LLCs) were used for tumor inoculation in-vivo. The inhibition of p75 receptor expression on the protein level was confirmed by western blot analysis using protein lysates of p75 shRNA transfected tumor cells and anti-TNFR2/p75 antibody (Cell Signaling Technology, Danvers, MA). The specificity of the bands was confirmed by positive and negative controls recommended by anti-TNFR2/p75 antibody manufacturer (Cell Signaling Technology).

Tumor inoculation

Cultured LLCs and p75KD/LLCs were harvested, counted and checked for viability using trypan blue exclusion. Cells were re-suspended in 100 μl PBS at a concentration of 5×10^5 cells per mice, then mixed with 100 μl of Growth factor reduced and phenol free Matrigel (BD Biosciences, San Jose, CA) and injected subcutaneously (SC) in the right flanks of mice. All tumor inoculations performed on the same day consisted of two major groups of mice: WT/LLC consisted of WT mice that were injected with non-transfected LLCs (n = 15) and p75KD/LLC consisted of WT mice that were injected with stably transfected (≥ 90%) p75KD/LLCs (n = 15). Tumor growth was monitored on a daily basis post-inoculation. When tumors became palpable on day 7 we measured the long and short axes of each tumor using electronic calipers. The volume was calculated using the formula, $V = 0.52xLxW^2$; where L and W denote larger and shorter diameter, respectively.

Recombinant murine TNF and intra-tumoral injections

Intra-tumoral injection of mouse recombinant murine (rm) TNF-α (eBiosciences, San Diego, CA) was initiated on day 8 post tumor inoculation. TNF-α was administered at a dose of 12.5 ng/gram of mouse weight twice daily by intra-tumoral injections. TNF-α was diluted to a stock concentration of 0.5 μg/μl and then it was diluted to 1:16 for intra-tumoral injections. No TNF-α group was injected with normal saline (0.9% Sodium Chloride). Tumor volumes and body weights were monitored daily and tumor weights were measured on day 15 post-inoculation (also,

day 8 after the first intratumoral injection and day 2 after the last intratumoral injection), when the experiment was terminated due to large size of tumors in WT/LLC group injected with rmTNF.

Tissue collection and histology staining

Body weights for each mouse were monitored before and after exogenous TNF-α injection. By day 8 after initiation of rmTNF injections, the entire study was terminated using pentobarbital based euthanasia solution due to ethical reasons, as tumor growth was rapid in the WT/LLC+rmTNF group. In addition, severe tumor necrosis in the p75KD/LLC+rmTNF group, rendered tumor volume measurements inaccurate. At the end of the study, all mice were euthanized and body weights with and without tumors, tumor weights and tumor volumes were measured. Tumors were bisected completely and divided into three parts for paraffin and OCT embedding (OCT compound, Tissue-Tek, Torrance, CA) and snap frozen tissue. To evaluate possible systemic toxicity we also collected spleens, both femurs after euthanasia and obtained blood smears to analyze peripheral blood (PB) before euthanasia. Femurs were paraffin embedded through the long axis to obtain longitudinal bone and bone marrow sections. Paraffin embedded sections of tumors, spleen and femurs were processed for Hematoxylin and Eosin (H&E) staining while spleen and femur paraffin sections were also stained for Periodic Acid Schiff staining (PAS) using manufacturer's protocol (Electron Microscopy Sciences, Hatfield, PA).

Immunohistochemistry

Frozen tissue sections (6–8 μm thick) of tumors were fixed in cold acetone (4°C) for 10 minutes [13,20] and processed for immunoflourescent staining. Topro-3 nuclear staining (Life Technologies) was used in conjunction with all immunoflourescent staining to visualize cell nuclei. To evaluate apoptosis and tumor angiogenesis at the border-zone between tumor tissue and surrounding tissue (peri-tumor), sections were triple stained with ApopTag Fluorescein In Situ Apoptosis TUNEL Kit (Millipore, Billerica, MA) anti-CD31 antibody (BD Pharmingen, San Jose, CA) along with Alexa Fluor 555 goat anti-rat secondary (Life Technologies) and Topro-3. The peri-tumoral and tumor area were identified by H&E staining of adjacent sections.

Imaging and analysis

Tumors, spleens, femurs and PB smears from all treatment groups stained for H&E and PAS staining were analyzed by two pathologists blind-folded to the treatment conditions. All immuno-flourescent stained slides were analyzed using Laser Scanning Confocal Microscope (ZEISS). Expression of CD31 and TUNEL staining were evaluated in at least 4–5 animals/group using Image J program (v1.40, NIH) by measuring mean pixel intensity in 7–8 separate visual fields of 176,400 μm^2 per mouse (×20 images). Results were plotted as a graph between mean intensity (pixels) and treatment groups of mice.

Statistical analysis

All results were expressed as mean + SEM and plots obtained. Statistical analysis was performed on the data by one-way ANOVA (Stat View Software, SAS Institute Inc., Middleton, MA). Differences were considered significant at $P < 0.05$.

Mathematical model

An ordinary differential equation system of the hypothesized biological dynamics was implemented in Matlab and solved numerically using ODE23s. Viable tumor cells (V) grow logistically with rate α bound by the host vascular carrying capacity (K). Viable cells become necrotic (N) with rate β. Necrotic cells may be cleared with rate δ. Necrotic cells produce TNF (F) with rate θ, which decays with rate ω and increases with rate ε due to rmTNF injections on treatment days t^T. TNF stimulates vasculature formation via p75 receptor in host stromal cells with rate η_h and through p75 in viable tumor cells with rate η_c. Non-physiologic vasculature collapses with rate φ. TNF induces cell death with rate γ, of which a fraction ζ becomes necrotic. In p75KD/LLC TNF response goes exclusively through the p55 pro-apoptotic pathway ($\gamma = \gamma + \eta_c$) and angiogenesis stimulation through the p75 pathway is shut off ($\eta_c = 0$). This yields the following system of coupled ordinary differential equations:

$$\frac{dV}{dt} = \overbrace{\alpha V\left(1-\frac{V}{K}\right)}^{\text{logistic growth}} - \overbrace{\beta V}^{\text{viable cells becoming necrotic}} - \overbrace{fFV}^{\text{cell death through TNF and p55}}$$

$$\frac{dN}{dt} = \overbrace{\beta V}^{\text{viable cells becoming necrotic}} - \overbrace{\delta N}^{\text{clearance}} + \overbrace{\zeta fFV}^{\text{fraction of p55 death becoming necrotic}}$$

$$\frac{dF}{dt} = \overbrace{\theta N}^{\text{production by necrotic cells}} - \overbrace{\omega F}^{\text{decay}} + \overbrace{\delta_{t,t_T}\varepsilon}^{\text{treatment}}$$

$$\frac{dK}{dt} = \overbrace{\eta_h F}^{\text{endogenous stimulation; host cells}} + \overbrace{gFV}^{\text{stimulation through viable cells p75}} - \overbrace{\varphi K\left(\frac{K}{K_0}\right)^4}^{\text{vasculature renormalization}}$$

$$f = \begin{cases} \gamma, & \text{for intact LLC} \\ \gamma + \eta_c, & \text{for p75KD/LLC} \end{cases} \qquad g = \begin{cases} \eta_c, & \text{for intact LLC} \\ 0, & \text{for p75KD/LLC} \end{cases}$$

where $\delta_{t,t}{}^{T}$ is a Kronecker delta enabling TNF injections with strength ε during treatment time t^T. $V(0) = V_0 = 132\ mm^3$–$246\ mm^3$, the sizes of the palpable tumors at day 7 post inoculation, $N_0 = F_0 = 0$, and $K_0 = 400\ mm^3$.

Results

Intra-tumoral TNF injections do not change mice body weight in spite of inducing tumor necrosis

In this study we used p75KD/LLC cells that were generated and characterized in our laboratory earlier [13]. p75KD/LLC cells were replated, propogated and used for our study after re-confirming knockdown of p75 receptor on the protein level (Fig. 1A). p75KD/LLC cells from group #1 and #3 (Fig. 1A) were used for tumor inocualtion in this study. Intact LLCs and p75KD/LLCs were inoculated into mice flanks (1×10^6 cells). All 30 animals developed comparable size flank tumors by day 7 (volume 257 ± 20.8 mm^3) and there was no difference in the tumor volumes between intact LLC vs. p75KD/LLC (242 ± 34.9 mm^3 vs. 263 ± 29.9 mm^3, respectively, P = NS). Starting on day 8 post-inoculation tumor volumes were measured daily in the morning followed by intra-tumoral injections of either saline or rmTNF (12.5 ng/g of mouse weight) twice a day for 6 days. No mortality or distress was observed in any of the four treatment groups. Over 15 days the body weights remained stable (at 22–24 gr/mouse) in all groups, including two groups that received intra-tumoral TNF injections (Fig. 1). This might suggest that local injection of low-dose recombinant murine TNF (rmTNF) is not likely to cause weight loss (i.e., cachexia/wasting syndrome) [21]. The study was terminated on day 8 after initiation of intra-tumoral injections due to large tumors (>1126 ± 293 mm^3 or >1.2 ± 0.34gr) in LLC+TNF group (Fig. 2A and 2B–C, red bars). These findings suggest that when both receptors are present, local low-

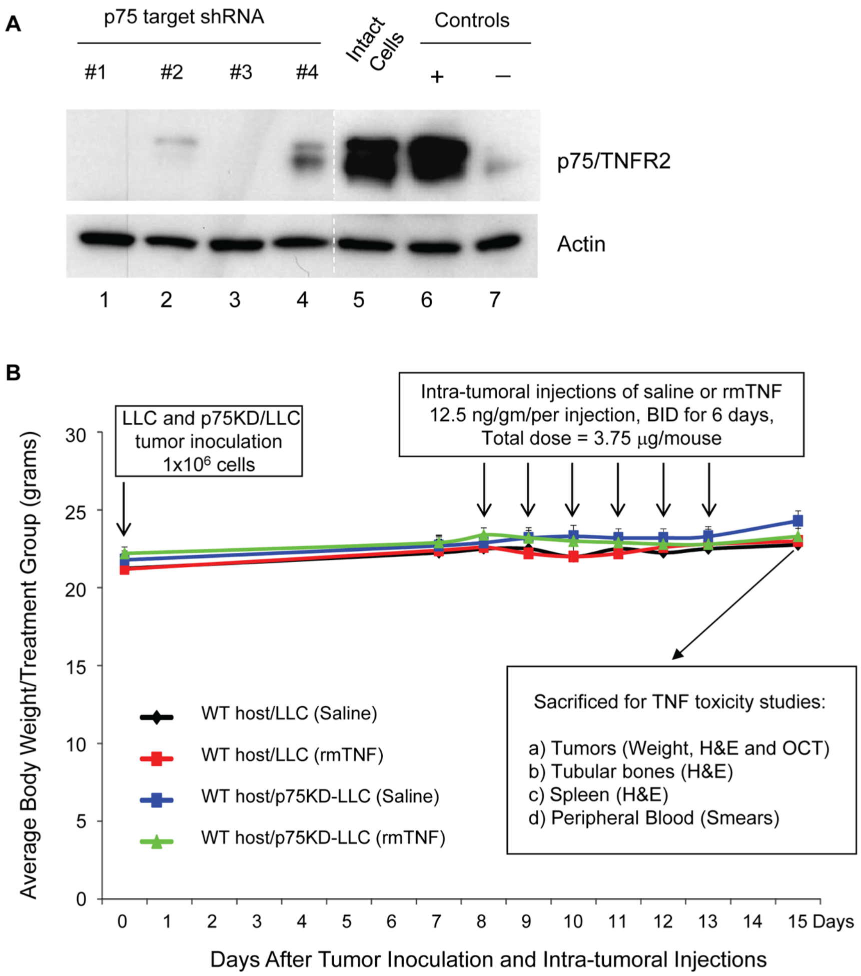

Figure 1. (A) Evaluation of p75 receptor expression in p75 shRNA transfected tumor cells by western blot analysis. Lanes 1, 2, 3 and 4 were transfected with various combinations of p75 target shRNA as previously described [13]. To confirm specificity of the bands we ran positive and negative control protein lysates recommended by the p75 antibody manufacturer. Actin expression was used as loading control. Compared to the expression of p75 receptor in intact tumor cells (lane 5) target sequences of p75 shRNA #1 and #3 showed no detectable expression of TNF receptor p75 (compare lane 5 to lanes 1 and 3). (B) Experimental design and body weight change over the course of the study. Intact LLC (LLC) and LLC with knockdown of TNFR2/p75 (p75KD/LLC) were inoculated into mice flanks (1×10^6 cells). LLC group consisted of WT mice that were injected with intact LLCs ($n = 15$) and p75KD/LLC consisted of WT mice that were injected with stably transfected (≥90%) p75KD/LLCs ($n = 15$). The two major groups WT host/LLC and WT host/p75KD/LLC were further divided into four groups: LLC minus (−) TNF consisted of WT mice inoculated with intact LLC that were injected with saline ($n = 5$), LLC plus (+) TNF consisted of WT mice with intact LLC that were injected with rmTNF ($n = 10$), p75KD/LLC-TNF consisted of WT mice with p75KD/LLC that were injected with saline ($n = 5$) and p75KD/LLC+TNF consisted of WT mice with p75KD/LLCs that were injected with rmTNF ($n = 10$). Tumor growth was monitored on a daily basis post-inoculation. Body weight data were plotted as a graph between tumor volume (mm^3) and time period after tumor inoculation for all groups. Tumors, including peri-tumoral stroma, were carefully bisected to make sure that tumor structure is intact and tumors were weighted. Tumors, femurs, spleens and peripheral blood were collected for histology staining to evaluate possible treatment toxicity and inflammatory responses.
doi:10.1371/journal.pone.0092373.g001

doses of rmTNF injections stimulate significant tumor growth [22,23] despite of some tumor necrosis (Fig. 2A and Fig. 3C).

Low-dose exogenous rmTNF injections decrease tumor growth and induce 40–100% necrosis in p75KD/LLCs

There was no significant difference in tumor volumes (526 ± 191 mm^3 vs. 853 ± 463 mm^3, P = NS) or weights (0.55 ± 0.11gr vs. 0.78 ± 0.12gr, P = NS) between intact LLC and p75KD/LLC tumors injected with saline, 2 days after the last rmTNF injection (Fig. 2B–C). On day 15, compared to three control groups, p75KD/LLC tumors injected with rmTNF showed >2-5-fold decrease in tumor volumes ($P < 0.001$) and weights ($P < 0.0001$) (Fig. 2A–C). Moreover, only in rmTNF-injected p75KD/LLC tumors there was no statistical significant difference between tumor volumes (206 ± 26.5 mm^3 vs. 291 ± 105 mm^3, P = NS) before injections vs. end of study (Fig. 2B and C, magenta bars), strongly suggesting that blocking p75 expression in tumor tissue combined with administration of small dose of rmTNF can significantly inhibit aggressive LLC tumor growth. Contrarily, in the other control groups tumor volumes were increased 2.7-, 3.2- and 4.5- fold (before vs. end of the study, $P < 0.01$, $P < 0.0002$ and $P < 0.0001$, respectively) (Fig. 2B and C).

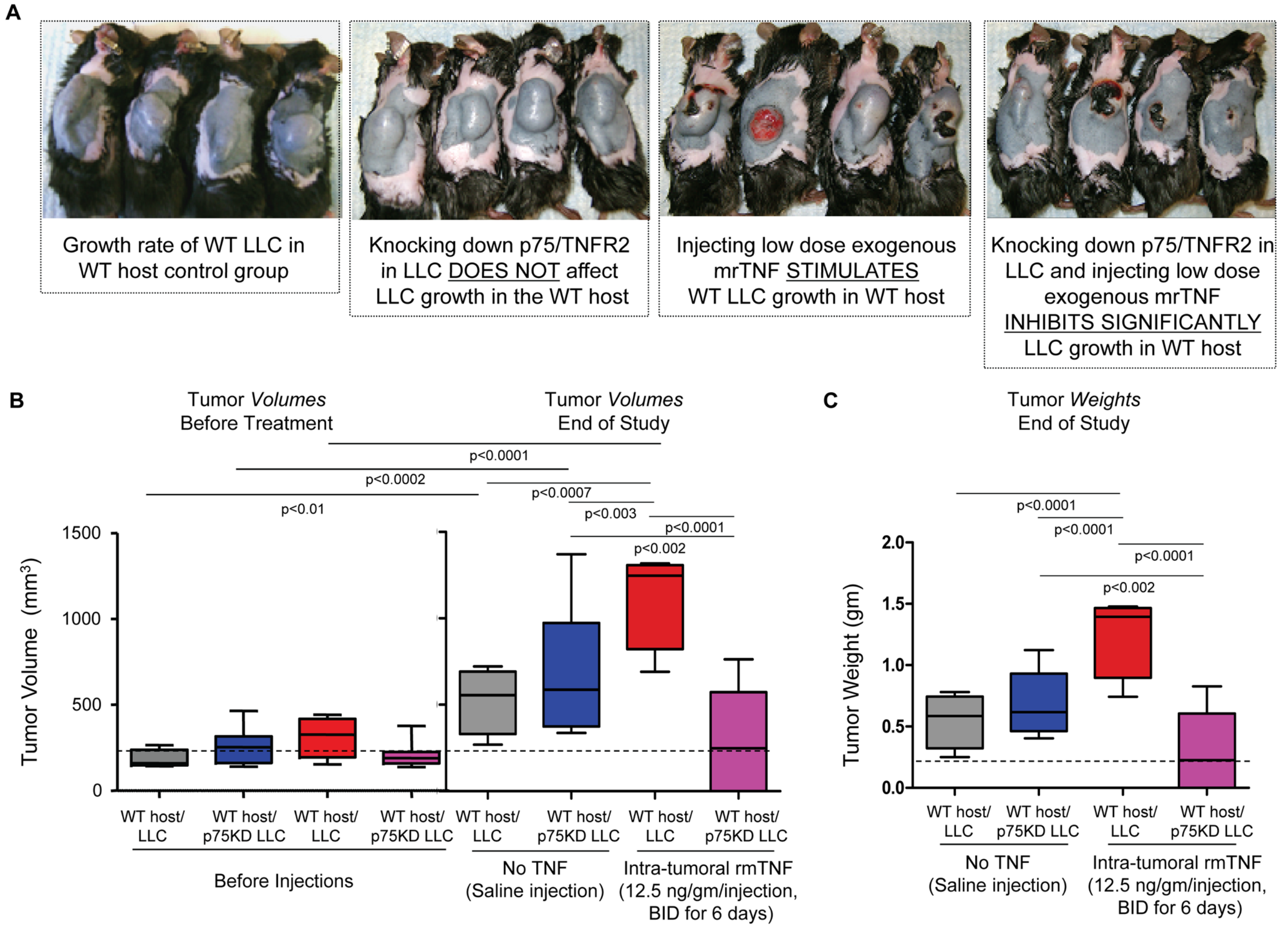

Figure 2. Flank tumor appearance at the end of the study and graphic representation of tumor volumes and weights. (A) Representative images of mice with flank tumors in (left to right) intact LLC in WT host injected with saline; p75KD/LLC in WT host injected with saline showing that knocking down p75/TNFR2 in LLC does not affect LLC growth in the WT host; intact LLC in WT host injected with rmTNF showing that injecting low dose exogenous rmTNF stimulates WT LLC growth in WT host; and p75KD/LLC in WT host injected with rmTNF showing that knocking down p75/TNFR2 in LLC and injecting *very* low dose of exogenous rmTNF significantly inhibits LLC growth in WT host. **(B)** Flank tumor volumes collected from 5–10 mice/treatment group before the first rmTNF injection (day 8 after initial tumor inoculations) and at end of the study (day 15 after initial inoculations). **(C)** Graphic representation of completely bisected flank tumor weights data collected from 5–10 mice/treatment at end of the study.
doi:10.1371/journal.pone.0092373.g002

Histological evidence of tumor necrosis and inhibition of tumor growth in p75KD/LLC tumors injected with rmTNF

WT/LLC saline-injected tumors exhibited ~95% viable neoplastic growth with small foci of peripheral necrosis (6 ± 1.4%). The inoculated LLC tumors displayed poorly differentiated carcinoma morphology with solid architectural pattern, highly pleomorphic, hyperchromic tumor cells and increased mitotic activity 22.5 ± 4.5 per high power field (HPF) magnification (Fig. 3A and Table 1, top row). Stromal component was depleted and tumors demonstrated high vascularity.

p75KD/LLC saline-injected tumors were characterized mostly by viable tumor infiltrating the surrounding adipose tissue and skeletal muscle. The mitotic count was similar to the control group (22.3 ± 2.2/HPF) (Fig. 3B and Table 1, second row from the top). There were foci of intra-parenchymal hemorrhage and ~5–10% of tumor tissue showed coagulative type necrosis predominantly in the periphery of tumors with minimal neutrophilic reaction.

WT/LLC+TNF tumors showed significant dermal involvement and were characterized by neoplastic growth similar to the control group with very high mitotic count 43.5 ± 21/HPF. There were large areas 36.5 ± 4.6 of coagulative type necrosis, predominantly on the periphery, with abundant cellular apoptosis, albeit with absence of inflammatory reaction (Fig. 3A and Table 1, third row from the top).

p75KD/LLC tumors injected with rmTNF showed a range of morphologic findings that varied from entirely necrotic tumors in 1/3 of the samples, to approximately half necrotic 44.7 ± 13.5% with mild to moderate acute inflammatory response and granulation tissue formation (Fig. 3D and Table 1, bottom row). Distribution of necrotic areas in partially viable tumors was variable (central and peripheral). In partially viable tumors there was a significant decrease in the mitotic count in this group −18 ± 10.4/HPF (Table 1, bottom row).

Increased tumor cell and tumor vasculature apoptosis in p75KD/LLC tumors injected with rmTNF

Compared to control groups the TUNEL staining, representing apoptosis, was increased >2.5-fold in p75KD/LLC tumors

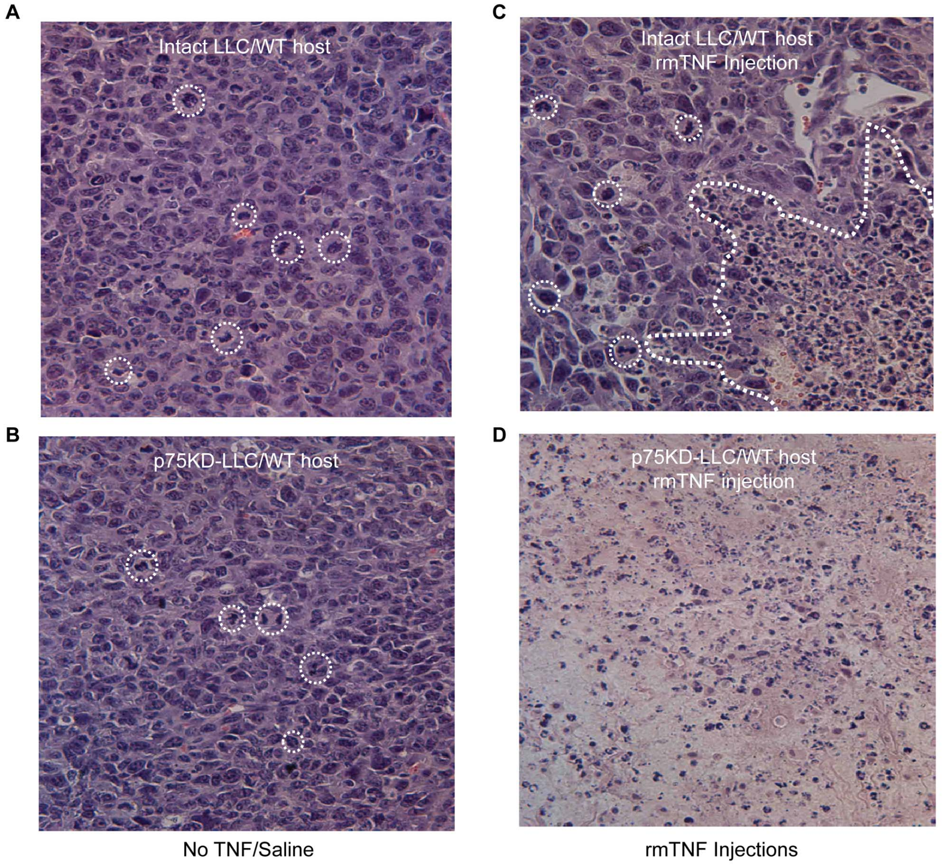

Figure 3. Tumor histology. Representative images of tumor H&E stained sections from four treatment groups, light microscopy at ×40 magnification. **(A)** Intact LLC in WT host injected with saline - viable tumor composed of highly pleomorphic malignant epithelial cells and brisk mitotic index. No necrosis seen. **(B)** p75KD/LLC in WT host injected with saline - viable carcinoma showing high mitotic activity and rare apoptotic bodies. **(C)** Intact LLC in WT host injected with rmTNF - partially viable carcinoma with focal necrosis and mild inflammatory change. **(D)** p75KD/LLC in WT host injected with rmTNF - massively necrotic tumor with no viable cells present. There was moderate acute inflammatory infiltrate in the tumor tissue. Please note that dotted circles in *A*, *B* and *C* indicate representative mitotic tumor cells.
doi:10.1371/journal.pone.0092373.g003

injected with rmTNF ($P < 0.04$ vs. all other groups) (Fig. 4A–E). There was also ~2-fold increase ($P < 0.003$) in apoptosis in p75KD/LLC+rmTNF tumors vs. intact LLC and p75KD/LLC tumors injected with saline (Fig. 4A, B and E). There was a small but statistically significant 25% increase ($P < 0.04$) in double TUNEL/CD31 positive cells, indicating tumor endothelial cell (EC) apoptosis, in p75KD/LLC+rmTNF vs. intact LLCs+rmTNF (Fig. 4 C, D and F). This suggests that knocking down only TNFR2/p75 in tumor cells combined with very small intratumoral rmTNF injections affect viability of tumor cells and tumor associated ECs and, to a lesser degree, in mice implanted with intact LLC.

Table 1. Summary of tumor tissue morphologic assessment.

Pathology Evaluation	Mitosis (HPF)	Necrosis	Inflammation	Microscopy General
**Treatment Groups**				
Intact LLC – WT host Saline Injection	22.5 ± 4.5	6 ± 1.4	None	Large, highly proliferative, aggressive tumors, no necrosis
p75KD/LLC – WT host Saline Injection	22.3 ± 2.2	13.3 ± 6	None	Large, highly proliferative, aggressive, mostly viable tumors, insignificant focal apoptosis and necrosis
Intact LLC – WT host mrTNF Injection	43.5 ± 21	36.5 ± 4.6	Very mild focal	Large tumor, highly mitotic – up to 106 mitotic bodies/HPF, aggressive, mild apoptosis and necrosis, mostly viable
p75KD/LLC – WT host mrTNF Injection	18 ± 10.4	44.7 ± 13.5	Mild to Moderate	No tumor left in 1/3 of the samples, while 2/3 of tumors are 40–60% necrotic, with signs of acute and chronic inflammation

Morphological findings in four treatment groups including - mitotic counts, area of necrosis, inflammatory infiltrate and major morphological findings. To avoid inter-observer variability a single clinical pathologist who was blinded to treatment conditions had evaluated H&E and PAS stained slides for all four treatment groups.
doi:10.1371/journal.pone.0092373.t001

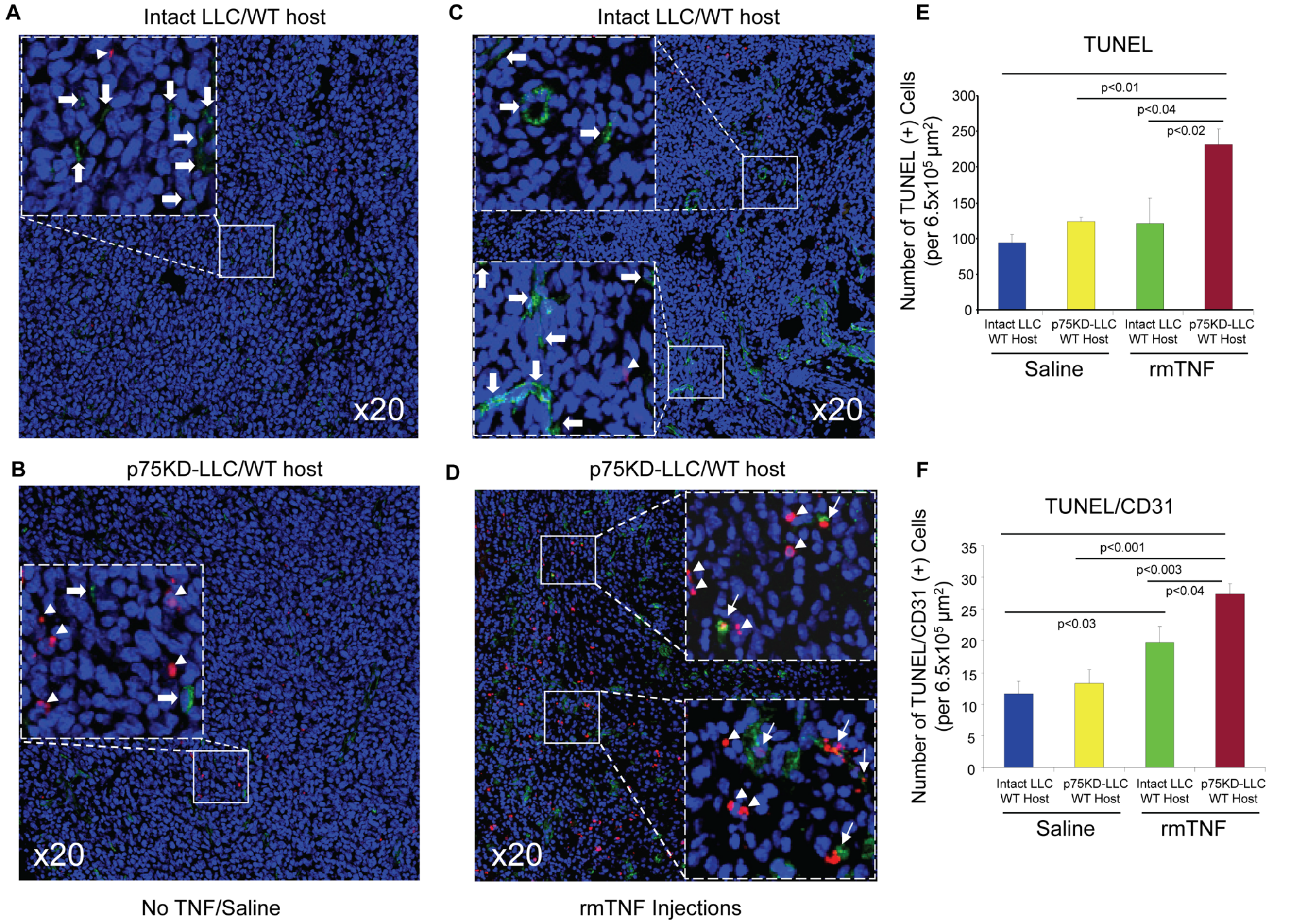

Figure 4. Evaluation of tumor and EC apoptosis. Apoptosis and tumor angiogenesis was evaluated in tumor tissues by triple immunostaining with terminal transferase dUTP nick end labeling (TUNEL), CD31 and Topro-3. The tumor area was identified by H&E staining of adjacent sections. **(A–D)** Representative images of triple-immunostained tumors for TUNEL (red), CD31 (green) and Topro-3 (blue); Insets identified by dashed squares in A–D indicate higher magnification of the selected areas in solid squares. Arrowheads indicate TUNEL (+) cells (red); block arrows indicate CD31 (+) cells (green) and arrows indicate double TUNEL/CD31 (+) cells (red/green and yellow). **(E)** Quantification and graphic representation of only TUNEL (+) cells in all four treatment groups. **(F)** Quantification and graphic representation of double TUNEL/CD31 (+) cells in all four groups.
doi:10.1371/journal.pone.0092373.g004

Mathematical model qualitatively reproduces experimental tumor growth dynamics

Numerical simulations of the mathematical model of tumor volume evolution of intact LLC injected with saline and p75KD/LLC+rmTNF are in excellent agreement with the experimental data (Fig. 5). Simulation of tumor growth in p75KD/LLC+rmTNF reproduces the initial total tumor volume (*V*+*N*; *viable+necrotic cells*) plateau that is followed by tumor growth and subsequent decrease in tumor volume (Fig. 5D, solid black curve). Low-dose injections of rmTNF stimulate transient angiogenesis and vasculature carrying capacity (*K*) increase yielding an increase in viable cells (*V*). Larger TNF concentration (*F*) and larger tumor volume are accompanied by an increase in necrotic mass (*N*). Necrosis yields further endogenous TNF production and subsequent induction of cell death and necrosis. The necrotic population and growing TNF concentration reverse tumor growth and decrease tumor volume. rmTNF injections in intact LLC yield sustained tumor growth supported by pro-angiogenic responses of host stromal cells and p75 proficient LLC. At later times, however, increase in TNF-induced apoptosis and accumulating necrotic mass slows down tumor growth (Fig. 5D) predicting a balance of cell proliferation and cell death. At day 15 post-inoculation, 2 days after stopping rmTNF injections, the simulated intact LLC tumor contains $\sim$19% necrosis compared to $\sim$39% in p75KD/LLC+rmTNF (Fig. 5, red curves) in agreement with experiments (Table 1).

Low dose rmTNF injection does not induce bone marrow or spleen toxicity and no change is detected in the peripheral blood

In intact LLC and p75KD/LLC groups with no TNF, bone marrow (BM) and spleen showed normal histology (Fig. 6A, B). In intact LLC+rmTNF and p75KD/LLC+rmTNF groups, there was a moderate to marked granulocytic hyperplasia, representing an inflammatory response or cytokine stimulation in the BM (Fig. 6B). Increased extramedullary hematopoiesis in red pulp was observed in the spleens of all mice injected with rmTNF (Fig. 6E, F). Importantly, there was no histologic evidence of necrosis or cellular damage in the BM or the spleen. Peripheral blood (PB) smears did not show any evidence of inflammatory leukocytosis or morphologic difference between the control saline injection groups

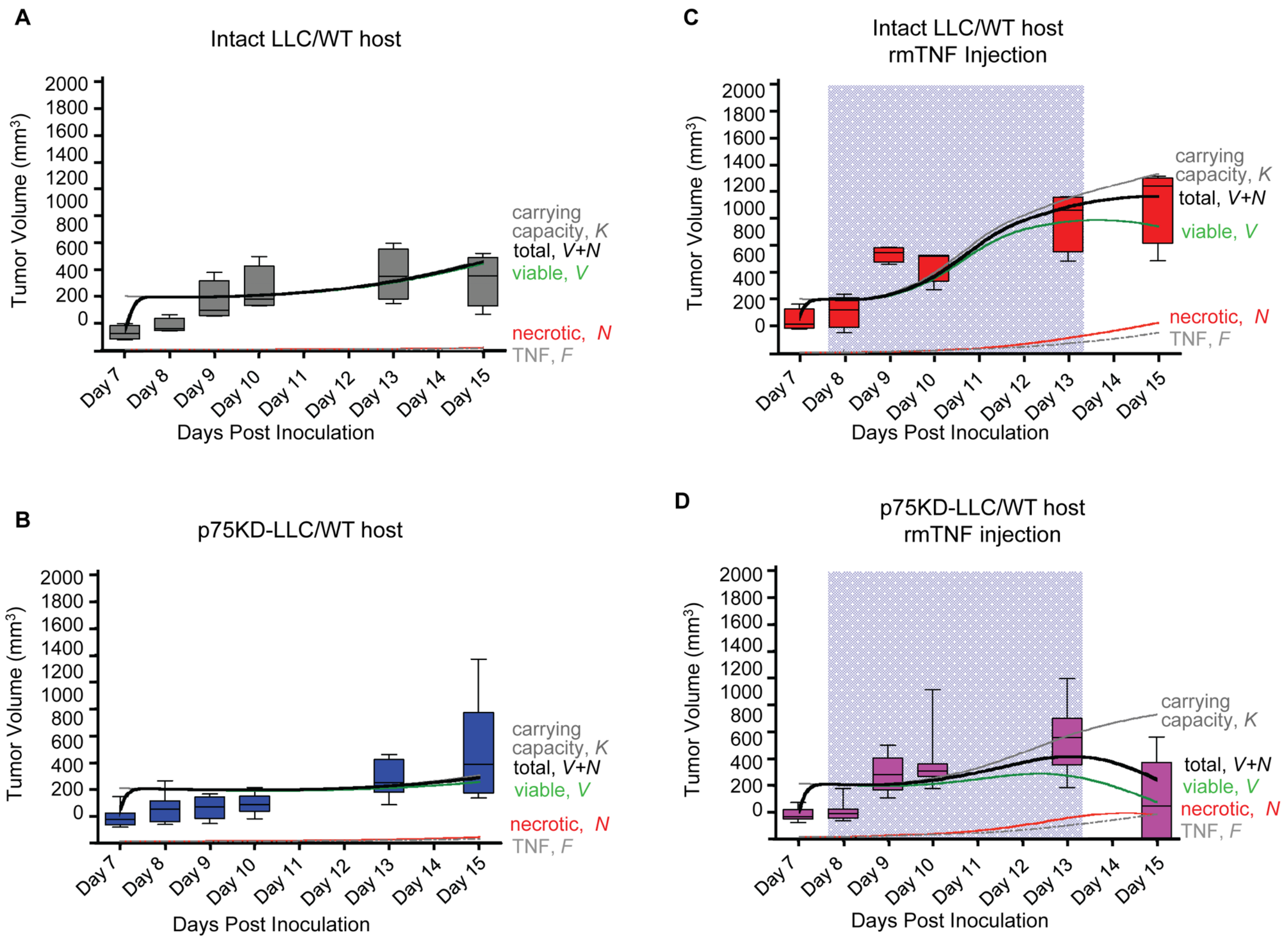

Figure 5. Numerical simulation results of the mathematical model. Total tumor populations (black solid curves) made up of viable tumor cells (*V*, green) and necrotic cells (*N*, red) quickly approach vascular carrying capacity (*K*, grey) and continue to growth after carrying capacity increase through host angiogenic response to necrosis-secreted TNF (*F*, grey dot-dashed). **(A)** Slowly emerging necrotic cells secrete TNF (*F*, grey dot-dashed) that stimulates transient angiogenesis through host cells and p75-competent cancer cells. Intact LLC tumor volume closely follows the increasing carrying capacity. Final necrotic tumor fraction is ~2%. Experimentally measured tumor volumes (grey box plots) shown for model validation. **(B)** p75KD-LLC tumor growth dynamic mimic intact LLC growth. Smaller tumor growth due to impaired pro-angiogenic signaling through p75. Final necrotic tumor fraction is ~7%. Experimentally measured tumor volumes (blue box plots) shown for model validation. **(C)** Carrying capacity transiently increases through injection of rmTNF (*F*, grey dot-dashed, in blue highlighted time interval) initially stimulating tumor growth. Increase in necrotic mass limits tumor growth to below carrying capacity. Final necrotic tumor fraction is ~19%. Experimentally measured tumor volumes (red box plots) shown for model validation. **(D)** Carrying capacity transiently increases through injection of rmTNF (*F*, grey dot-dashed, in blue highlighted time interval) initially stimulating p75KD/LLC+rmTNF tumor growth and later dwarfing tumor growth through TNF-induced cell death and increasing necrosis. Final necrotic tumor fraction is ~39%. Experimentally measured tumor volumes (magenta box plots) shown for model validation. Model parameters: $\alpha = 10$, $\beta = 0.06$, $\gamma = 0.02$, $\zeta = 0.5$, $\delta = 6.2$, $\eta_h = 6$, $\eta_c = 0.025$ ($\eta_c = 0$ for p75KD/LLC+rmTNF), $\theta = 0.24$, $\varepsilon = 6$ ($\varepsilon = 0$ on non-treatment days), $\omega = 0.003$, $\varphi = 0.02$.
doi:10.1371/journal.pone.0092373.g005

vs. rmTNF treatment groups (Fig. S1A and B). Taken together, our data confirms the absence of tissue necrosis or cytologic damage in BM, spleen and PB, suggesting that small doses of intratumoral rmTNF injection in p75KD/LLC implanted mice did not induce systemic toxicity despite massive tumor necrosis (Fig. 3D).

Discussion

TNF can function as either an angiogenic or anti-angiogenic factor, depending on the specific disease settings [24–26]. Low doses of TNF promote tumor growth and progression while high doses of TNF have tumor inhibitory effects [22] (Fig. S2A and S2B). TNF receptors, p55 and p75, trigger divergent signaling pathways upon interaction with the ligand and lead to different biological outcomes [23]. Activation of the caspases and subsequent induction of apoptosis by TNF is an exclusive feature of p55 signaling [27]. On the other hand, p75 mediates TNF-induced survival and angiogenic effects [13]. Hence, the bias towards TNF-TNFR1/p55 signaling cascade is generally pro-apoptotic while signaling through TNFR2/p75 is pro-survival and pro-angiogenic. The role of TNFR2/p75 in tumor biology was further substantiated by increased accumulation of $CD4^+$ $CD25^+$ $FoxP3^+$ T regulatory cells (Tregs) that express $TNFR2^+$ but not $TNFR^-$ Tregs in the LLC tumor model [28]. Preferential accumulation of $TNFR2^+$ Tregs in tumor-infiltrating lymphocytes (TILs) may have significant clinical value as inhibition of TNFR2/p75 in this subset of TILs will render them more sensitive to administration of small exogenous doses of TNF, therefore inducing apoptosis and necrosis of these cells. To take advantage of this phenomenon,

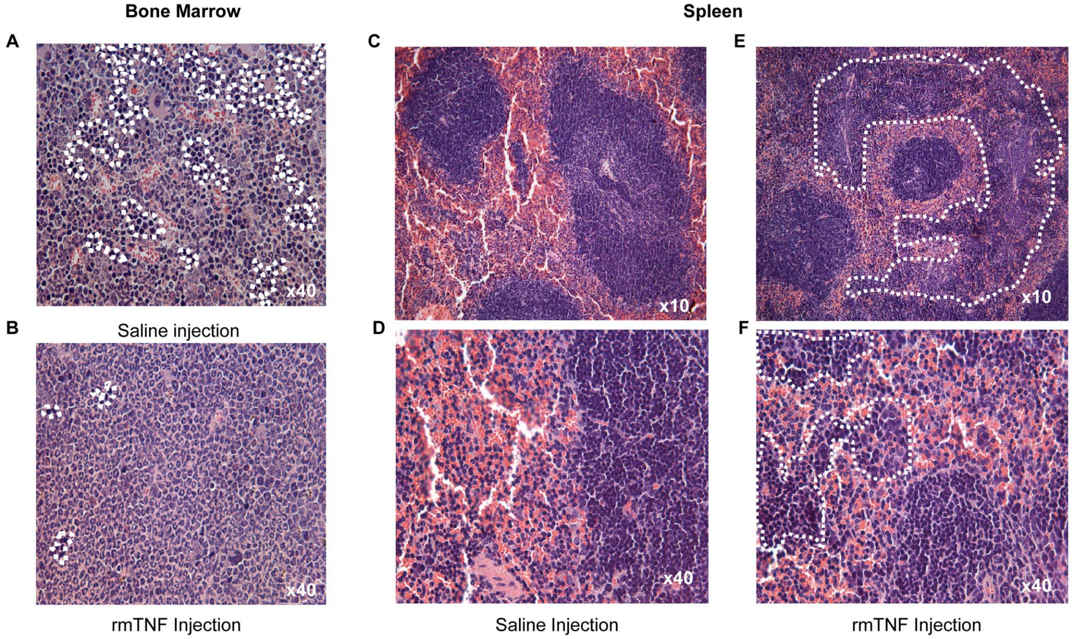

Figure 6. Evaluation of possible exogenous rmTNF toxicity in bone marrow and spleen. Representative images of H&E stained bone marrow and spleen tissue. **(A–B)** Bone marrow - There was granulocytic hyperplasia in the BM of mice with necrotic tumors in rmTNF-injected groups, reflected by a shift of the myeloid/erythroid ratio (~3:1 vs. ~8:1) in the BM (erythroid islands indicated within the encircled areas). **(C–F)** Spleen - There was a marked increase in extramedullary hematopoiesis in spleens of mice with necrotic tumors in rmTNF injected indicated by the cellularity within the encircled regions between normal lymphoid tissue (white pulp).
doi:10.1371/journal.pone.0092373.g006

we tested a method of selective TNFR2/p75 inhibition for cancer treatment. The main biological processes affected by TNF-TNFR1/p55 and TNF-TNFR2/p75 interactions in the presence of low or high doses of TNF are depicted in Fig. S2A and S2B. The hypothetical Fig. S2A shows that inhibition of TNFR2/p75 expression or signaling should have multifaceted anti-tumor effects irrespective of low or high doses of TNF. Numerical solutions of a mathematical formalization of the opposing pro-angiogenic and cytotoxic functions of TNF (Fig. 5) qualitatively reproduce the experimentally observed tumor growth dynamics and lend further support to the therapeutic promise of TNFR2/p75 inhibition. Fully calibrated, the model can simulate the response of tumors to TNF injections and will help predict optimal TNF treatment schedules to completely eradicate viable tumor cells.

Our earlier published findings in wild type (WT), p55KO, p75KO and double p55KO/p75KO murine LLC and B16 melanoma tumor models suggest that the absence of p75 signaling in tumor tissue in vivo may deliver a "double hit" by affecting survival and function of ECs and of tumor cells, while antecedent high levels of TNF (due to an ongoing tumor necrosis) in tumor tissue could have self-destructive effect [13]. We also found, that the absence of p75 in the host tissue (p75KO mice) has more significant inhibitory effect on the expression of several pro-angiogenic and pro-survival molecules in the tumor tissue as compared to the absence of p55 (p55KO mice) [13].

It is noteworthy to discuss briefly the potential clinical applications and the market value of cytokine-based therapies. It is significantly underscored by the fact that more than 120 companies are developing over 270 new therapies that either are cytokines, mimic cytokines, or inhibit cytokines and/or cytokine receptors. The usefulness of cytokine-based therapies in the clinical setting is underscored by the fact that several of these products are currently attaining over $1 billion in annual sales. Some of these therapies represent established markets, but there are numerous additional opportunities being pursued by pharmaceutical companies.

TNF family-based therapies on the market and/or being developed could be divided into three major groups. First, there are inhibitors of the TNF family, such as monoclonal antibodies infliximab, adalimumab, or a circulating receptor fusion protein such as etanercept, that are predominantly used for the treatment of autoimmune diseases, i.e., rheumatoid arthritis (RA) or ankylosing spondylitis. Infliximab and Adalimumab are monoclonal antibodies, whereas Etanercept is a recombinant human fusion protein that consists of two soluble p75 receptors and the Fc portion of human IgG1 [19,29,30]. Both monoclonal antibodies and soluble receptors mediate their effect via binding with high specificity and affinity to soluble and membrane-bound TNF and block the interaction of TNF with the p55 and p75 receptors, hence decreasing bioavailability of TNF, thereby neutralizing to a certain extent the biological activities of TNF [17–19].

Then there is a specific inhibitor of the soluble B-lymphocyte stimulator (BLyS) cytokine, which has been implicated in the pathogenesis of systemic lupus erythematosus (SLE). A fully human monoclonal antibody that binds to and inhibits receptor activator of nuclear factor kappa-B ligand (RANKL), such as denusumab, is used to treat osteoporosis or bone metastasis. In this group the CD30 inhibitors, such as brentuximab and vedotin, are used to treat anaplastic large T-cell systemic malignant lymphoma or Hodgkin's lymphoma.

Second, there are TNF family inhibitors with anti-cancer activity currently on the market: (a) Anthera Pharmaceuticals' Blisibimod - is a selective antagonist of B-cell activating factor (BAFF, also known as B-lymphocyte stimulator or BLyS), (b) Eli Lilly's LY2127399, a human monoclonal antibody that neutralizes B-cell activating factor (BAFF), for use in combination with

bortezomib in patients with previously-treated multiple myeloma. In addition, LY2127399 is also in Phase III evaluation as a potential treatment for RA and SLE.

Third, there are two other approaches for inhibition of TNF expression and inhibition of TNF oligomerization. These include Thalidomide that is currently being used for treatment of multiple myeloma [31] and Pentoxifylline is used to treat leg pain due to poor circulation [32]. Hence, these agents may be useful for the treatment of some cancers (for prevention purposes only) where TNF is a distinct etiologic factor (e.g., inflammation) [33–35]. In this case these agents are most likely to be effective in early stages of tumorigenesis (initiation and promotion). Another group has identified a small molecule inhibitor that promotes disassociation of the homotrimeric TNF [36]. However, the effectiveness of these later approaches remains to be tested in the clinical setting(s).

Finally, human recombinant TNF (hrTNF) in combination with Melphalan is being used for treatment of advanced melanoma and inextirpable soft tissue sarcomas using isolated limb perfusion [14]. Despite of unmatched anticancer effects this approach may also induce excessive side effects such as septic shock-like syndrome, massive necrosis of non-tumor tissues and systemic inflammatory response [15,16]. In addition to the various side effects observed with these treatments such as development of lymphomas, greater predisposition towards infection, re-exacerbation of latent tuberculosis, and problems related to autoimmunity [30,37], systemically administered agents that affect the immune system most likely may/should not be used for cancer treatment as TNF is also needed for the proper functioning of the immune system. Complete or significant systemic suppression of TNF effects over a long period is likely to prove harmful.

As striking as it may seem, none of the TNF-based therapies indicated above are directed towards inhibiting the interaction between ligand TNF and one of its receptors, specifically TNFR2/p75, therefore blocking downstream to this specific TNF receptor 2 signaling pathways. To the best of our knowledge, there is no precedent in the available literature that proposes development of cancer treatment based on selective blocking of one of the TNF receptors.

In summary, the data in this manuscript represents a number of innovative components and an entirely new paradigm for cancer treatment. First, blocking of TNFR2/p75 signaling either in the tumor stroma (our earlier published work) [13] and/or the tumor cells themselves (data in this manuscript) leads to tumor regression. This approach inhibits selectively the pro-survival and pro-angiogenic TNFR2/p75. This fosters pro-death and anti-angiogenic signaling through the remaining cytotoxic TNFR1/p55, especially when apoptotic and necrotic tumor cells produce more TNF. In fact, the name tumor necrosis factor speaks for itself – the more tumor cells die, the more endogenous TNF will be produced locally in the tumor tissue, and in the absence of the signaling through p75 most of TNF signaling will go through p55 (pro-apoptotic receptor), hence causing more tumor and endothelial cell death (Fig. 3A–C and Fig. 4A–F). We believe, this is a promising approach to induce tumor cell death and diminish tumor blood supply with an anticipated reduction in undesired toxicity that is seen with the use of exogenous TNF in the ILP treatment regimens [14–16]. Second, the approach of p75 receptor signaling inhibition within the tumor is not targeted towards a specific cell type (tumor cells, ECs, stromal cells, etc). If successful, this ‘‘cell blind’’ approach may shift the paradigm of biological methods for cancer treatment. Although not explicitly discussed in this manuscript, the studies are underway to validate this prediction.

Supporting Information

Figure S1 Representative Images of Peripheral Blood Smears for all Groups. (A) At ×40 magnification a few neutrophils and/or basophils are noted. **(B)** ×10 magnification, dotted square indicates area of the ×40 mag. in A. The PB did not reveal any morphologic difference between the four treatment groups.
(EPS)

Figure S2 The main biological processes affected by TNF-TNFR1/p55 and TNF-TNFR2/p75 interactions in the presence of low or high doses/concentrations of TNF. (A) Biological processes in WT cells in the presence of low TNF dose/concentration. **(B)** Hypothetical diagram of the biological processes in TNFR2/p75 tumor cells in the presence of low or high TNF dose/concentration shows that inhibition of TNFR2/p75 expression or signaling should have multifaceted anti-tumor effects irrespective of low or high doses of TNF.
(EPS)

Author Contributions

Conceived and designed the experiments: DAG. Performed the experiments: SPS SB JS. Analyzed the data: SPS SB JS AP DS HE. Contributed reagents/materials/analysis tools: JC XY RK. Wrote the paper: SPS DAG.

References

1. Mirsadraee S, Oswal D, Alizadeh Y, Caulo A, van Beek E Jr (2012) The 7th lung cancer TNM classification and staging system: Review of the changes and implications. World journal of radiology 4: 128–134.
2. Jemal A, Bray F, Center MM, Ferlay J, Ward E, et al. (2011) Global cancer statistics. CA: a cancer journal for clinicians 61: 69–90.
3. Abdullah LN, Chow EK (2013) Mechanisms of chemoresistance in cancer stem cells. Clinical and translational medicine 2: 3.
4. Gatenby RA, Silva AS, Gillies RJ, Frieden BR (2009) Adaptive therapy. Cancer research 69: 4894–4903.
5. Botchkina GI, Meistrell ME 3rd, Botchkina IL, Tracey KJ (1997) Expression of TNF and TNF receptors (p55 and p75) in the rat brain after focal cerebral ischemia. Molecular medicine 3: 765–781.
6. Leeuwenberg JF, van Tits LJ, Jeunhomme TM, Buurman WA (1995) Evidence for exclusive role in signalling of tumour necrosis factor p55 receptor and a potentiating function of p75 receptor on human endothelial cells. Cytokine 7: 457–462.
7. MacEwan DJ (2002) TNF receptor subtype signalling: differences and cellular consequences. Cellular signalling 14: 477–492.
8. Kalthoff H, Roeder C, Brockhaus M, Thiele HG, Schmiegel W (1993) Tumor necrosis factor (TNF) up-regulates the expression of p75 but not p55 TNF receptors, and both receptors mediate, independently of each other, up-regulation of transforming growth factor alpha and epidermal growth factor receptor mRNA. The Journal of biological chemistry 268: 2762–2766.
9. Bhardwaj A, Aggarwal BB (2003) Receptor-mediated choreography of life and death. Journal of clinical immunology 23: 317–332.
10. Faustman D, Davis M (2010) TNF receptor 2 pathway: drug target for autoimmune diseases. Nature reviews Drug discovery 9: 482–493.
11. Li B, Vincent A, Cates J, Brantley-Sieders DM, Polk DB, et al. (2009) Low levels of tumor necrosis factor alpha increase tumor growth by inducing an endothelial phenotype of monocytes recruited to the tumor site. Cancer research 69: 338–348.
12. Meng Y, Beckett MA, Liang H, Mauceri HJ, van Rooijen N, et al. (2010) Blockade of tumor necrosis factor alpha signaling in tumor-associated macrophages as a radiosensitizing strategy. Cancer research 70: 1534–1543.
13. Sasi SP, Yan X, Enderling H, Park D, Gilbert HY, et al. (2012) Breaking the 'harmony' of TNF-alpha signaling for cancer treatment. Oncogene 31: 4117–4127.
14. Lejeune FJ, Lienard D, Matter M, Ruegg C (2006) Efficiency of recombinant human TNF in human cancer therapy. Cancer Immun 6: 6.
15. Hohenberger P, Latz E, Kettelhack C, Rezaei AH, Schumann R, et al. (2003) Pentoxifyllin attenuates the systemic inflammatory response induced during isolated limb perfusion with recombinant human tumor necrosis factor-alpha and melphalan. Annals of surgical oncology 10: 562–568.

16. Laurenzi L, Natoli S, Di Filippo F, Calamaro A, Centulio F, et al. (2004) Systemic and haemodynamic toxicity after isolated limb perfusion (ILP) with TNF-alpha. J Exp Clin Cancer Res 23: 225–231.
17. Keystone EC, Kavanaugh AF, Sharp JT, Tannenbaum H, Hua Y, et al. (2004) Radiographic, clinical, and functional outcomes of treatment with adalimumab (a human anti-tumor necrosis factor monoclonal antibody) in patients with active rheumatoid arthritis receiving concomitant methotrexate therapy: a randomized, placebo-controlled, 52-week trial. Arthritis Rheum 50: 1400–1411.
18. Maini R, St Clair EW, Breedveld F, Furst D, Kalden J, et al. (1999) Infliximab (chimeric anti-tumour necrosis factor alpha monoclonal antibody) versus placebo in rheumatoid arthritis patients receiving concomitant methotrexate: a randomised phase III trial. ATTRACT Study Group. Lancet 354: 1932–1939.
19. Bathon JM, Martin RW, Fleischmann RM, Tesser JR, Schiff MH, et al. (2000) A comparison of etanercept and methotrexate in patients with early rheumatoid arthritis. The New England journal of medicine 343: 1586–1593.
20. Goukassian DA, Qin G, Dolan C, Murayama T, Silver M, et al. (2007) Tumor necrosis factor-alpha receptor p75 is required in ischemia-induced neovascularization. Circulation 115: 752–762.
21. Bossola M, Pacelli F, Doglietto GB (2007) Novel treatments for cancer cachexia. Expert opinion on investigational drugs 16: 1241–1253.
22. Szlosarek P, Charles KA, Balkwill FR (2006) Tumour necrosis factor-alpha as a tumour promoter. European journal of cancer 42: 745–750.
23. Arnott CH, Scott KA, Moore RJ, Robinson SC, Thompson RG, et al. (2004) Expression of both TNF-alpha receptor subtypes is essential for optimal skin tumour development. Oncogene 23: 1902–1910.
24. Moore RJ, Owens DM, Stamp G, Arnott C, Burke F, et al. (1999) Mice deficient in tumor necrosis factor-alpha are resistant to skin carcinogenesis. Nature medicine 5: 828–831.
25. Arnott CH, Scott KA, Moore RJ, Hewer A, Phillips DH, et al. (2002) Tumour necrosis factor-alpha mediates tumour promotion via a PKC alpha- and AP-1-dependent pathway. Oncogene 21: 4728–4738.
26. Scott KA, Moore RJ, Arnott CH, East N, Thompson RG, et al. (2003) An anti-tumor necrosis factor-alpha antibody inhibits the development of experimental skin tumors. Mol Cancer Ther 2: 445–451.
27. Chen G, Goeddel DV (2002) TNF-R1 signaling: a beautiful pathway. New York, NYScience296: 1634–1635.
28. Chen X, Subleski JJ, Kopf H, Howard OM, Mannel DN, et al. (2008) Cutting edge: expression of TNFR2 defines a maximally suppressive subset of mouse CD4+CD25+FoxP3+ T regulatory cells: applicability to tumor-infiltrating T regulatory cells. J Immunol 180: 6467–6471.
29. Madhusudan S, Foster M, Muthuramalingam SR, Braybrooke JP, Wilner S, et al. (2004) A phase II study of etanercept (Enbrel), a tumor necrosis factor alpha inhibitor in patients with metastatic breast cancer. Clin Cancer Res 10: 6528–6534.
30. Nair B, Raval G, Mehta P (2007) TNF-alpha inhibitor etanercept and hematologic malignancies: report of a case and review of the literature. Am J Hematol 82: 1022–1024.
31. Harousseau JL (2006) Thalidomide in multiple myeloma: past, present and future. Future Oncol 2: 577–589.
32. Zabel P, Schade FU, Schlaak M (1993) Inhibition of endogenous TNF formation by pentoxifylline. Immunobiology 187: 447–463.
33. Aggarwal BB, Shishodia S, Ashikawa K, Bharti AC (2002) The role of TNF and its family members in inflammation and cancer: lessons from gene deletion. Curr Drug Targets Inflamm Allergy 1: 327–341.
34. Komori A, Yatsunami J, Suganuma M, Okabe S, Abe S, et al. (1993) Tumor necrosis factor acts as a tumor promoter in BALB/3T3 cell transformation. Cancer research 53: 1982–1985.
35. Suganuma M, Okabe S, Marino MW, Sakai A, Sueoka E, et al. (1999) Essential role of tumor necrosis factor alpha (TNF-alpha) in tumor promotion as revealed by TNF-alpha-deficient mice. Cancer research 59: 4516–4518.
36. He MM, Smith AS, Oslob JD, Flanagan WM, Braisted AC, et al. (2005) Small-molecule inhibition of TNF-alpha. New York, NYScience310: 1022–1025.
37. Hasan U (2006) Tumour necrosis factor inhibitors—what we need to know. N Z Med J 119: U2336.
